# Daratumumab-based quadruplet therapy for transplant-eligible newly diagnosed multiple myeloma with high cytogenetic risk

**DOI:** 10.1038/s41408-024-01030-w

**Published:** 2024-04-22

**Authors:** Natalie S. Callander, Rebecca Silbermann, Jonathan L. Kaufman, Kelly N. Godby, Jacob Laubach, Timothy M. Schmidt, Douglas W. Sborov, Eva Medvedova, Brandi Reeves, Binod Dhakal, Cesar Rodriguez, Saurabh Chhabra, Ajai Chari, Susan Bal, Larry D. Anderson, Bhagirathbhai R. Dholaria, Nitya Nathwani, Parameswaran Hari, Nina Shah, Naresh Bumma, Sarah A. Holstein, Caitlin Costello, Andrzej Jakubowiak, Tanya M. Wildes, Robert Z. Orlowski, Kenneth H. Shain, Andrew J. Cowan, Huiling Pei, Annelore Cortoos, Sharmila Patel, Thomas S. Lin, Smith Giri, Luciano J. Costa, Saad Z. Usmani, Paul G. Richardson, Peter M. Voorhees

**Affiliations:** 1 University of Wisconsin Carbone Cancer Center, Madison, WI USA; 2 Knight Cancer Institute, Oregon Health & Science University, Portland, OR USA; 3 Winship Cancer Institute, Emory University, Atlanta, GA USA; 4 University of Alabama at Birmingham Hospital, Birmingham, AL USA; 5 Dana-Farber Cancer Institute, Harvard Medical School, Boston, MA USA; 6 Huntsman Cancer Institute, University of Utah, Salt Lake City, UT USA; 7 Department of Medicine, University of North Carolina at Chapel Hill, Chapel Hill, NC USA; 8 Division of Hematology/Oncology, Department of Medicine, Mayo Clinic Arizona, Phoenix, AZ USA; 9 Icahn School of Medicine at Mount Sinai, New York, NY USA; 10 Myeloma, Waldenstrӧm’s and Amyloidosis Program, Simmons Comprehensive Cancer Center, UT Southwestern Medical Center, Dallas, TX USA; 11 Vanderbilt University Medical Center, Nashville, TN USA; 12 Judy and Bernard Briskin Center for Multiple Myeloma Research, City of Hope Comprehensive Cancer Center, Duarte, CA USA; 13 Department of Medicine, University of California San Francisco, San Francisco, CA USA; 14 Division of Hematology, The Ohio State University Comprehensive Cancer Center, Columbus, OH USA; 15 Division of Oncology & Hematology, Department of Internal Medicine, University of Nebraska Medical Center, Omaha, NE USA; 16 Moores Cancer Center, University of California San Diego, La Jolla, CA USA; 17 University of Chicago Medical Center, Chicago, IL USA; 18 Department of Lymphoma/Myeloma, The University of Texas MD Anderson Cancer Center, Houston, TX USA; 19 Department of Malignant Hematology, H. Lee Moffitt Cancer Center, Tampa, FL USA; 20 Division of Medical Oncology, University of Washington, Seattle, WA USA; 21 Janssen Research & Development, LLC, Titusville, NJ USA; 22 Janssen Scientific Affairs, LLC, Horsham, PA USA; 23 Division of Hematology & Oncology, Department of Medicine, University of Alabama at Birmingham, Birmingham, AL USA; 24 Memorial Sloan Kettering Cancer Center, New York, NY USA; 25 Levine Cancer Institute, Atrium Health Wake Forest Baptist, Charlotte, NC USA

**Keywords:** Targeted therapies, Myeloma

## Abstract

In the MASTER study (NCT03224507), daratumumab+carfilzomib/lenalidomide/dexamethasone (D-KRd) demonstrated promising efficacy in transplant-eligible newly diagnosed multiple myeloma (NDMM). In GRIFFIN (NCT02874742), daratumumab+lenalidomide/bortezomib/dexamethasone (D-RVd) improved outcomes for transplant-eligible NDMM. Here, we present a post hoc analysis of patients with high-risk cytogenetic abnormalities (HRCAs; del[17p], t[4;14], t[14;16], t[14;20], or gain/amp[1q21]). Among 123 D-KRd patients, 43.1%, 37.4%, and 19.5% had 0, 1, or ≥2 HRCAs. Among 120 D-RVd patients, 55.8%, 28.3%, and 10.8% had 0, 1, or ≥2 HRCAs. Rates of complete response or better (best on study) for 0, 1, or ≥2 HRCAs were 90.6%, 89.1%, and 70.8% for D-KRd, and 90.9%, 78.8%, and 61.5% for D-RVd. At median follow-up (MASTER, 31.1 months; GRIFFIN, 49.6 months for randomized patients/59.5 months for safety run-in patients), MRD-negativity rates as assessed by next-generation sequencing (10^–5^) were 80.0%, 86.4%, and 83.3% for 0, 1, or ≥2 HRCAs for D-KRd, and 76.1%, 55.9%, and 61.5% for D-RVd. PFS was similar between studies and superior for 0 or 1 versus ≥2 HRCAs: 36-month PFS rates for D-KRd were 89.9%, 86.2%, and 52.4%, and 96.7%, 90.5%, and 53.5% for D-RVd. These data support the use of daratumumab-containing regimens for transplant-eligible NDMM with HCRAs; however, additional strategies are needed for ultra-high–risk disease (≥2 HRCAs).

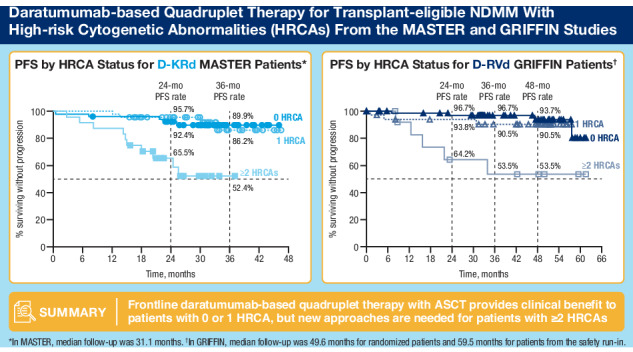

## Introduction

Daratumumab is a human IgGκ monoclonal antibody targeting CD38 with a direct on-tumor [1–4] and immunomodulatory [5–7] mechanism of action, demonstrating greater cytotoxicity toward multiple myeloma (MM) cells ex vivo compared with analogs of other CD38 antibodies [8]. Daratumumab is approved as a monotherapy and in combination with standard-of-care regimens for patients with relapsed or refractory MM and a part of combination therapy for patients with newly diagnosed MM (NDMM) [9].

Induction therapy with a triplet regimen consisting of a proteasome inhibitor, immunomodulatory drug, and dexamethasone is standard of care for transplant-eligible patients with NDMM [10]. Recent studies have examined the addition of daratumumab to these triplet regimens. The single-arm, phase 2 MASTER study (ClinicalTrials.gov Identifier: NCT03224507) evaluated daratumumab plus carfilzomib, lenalidomide, and dexamethasone (D-KRd) and demonstrated promising clinical efficacy in transplant-eligible patients with NDMM [11]. The primary analysis (median follow-up, 25.1 months) showed that minimal residual disease (MRD) negativity at the 10^–5^ threshold was achieved by 80% of D-KRd patients, as determined at the end of MRD-directed treatment [11]. The final analysis of MASTER was recently reported, and data continued to demonstrate a benefit of D-KRd in this setting in the overall population and suggested a possible pathway for treatment cessation among responding patients exhibiting sustained MRD negativity [12]. The randomized, phase 2 GRIFFIN study (ClinicalTrials.gov Identifier: NCT02874742) evaluated daratumumab plus lenalidomide, bortezomib, and dexamethasone (D-RVd) or lenalidomide, bortezomib, and dexamethasone (RVd) alone in transplant-eligible patients with NDMM [13]. The primary analysis (median follow-up, 13.5 months) showed that the rate of stringent complete response (CR) by the end of post-autologous stem cell transplant (ASCT) consolidation was significantly higher for D-RVd versus RVd (42.4% vs 32.0%; 1-sided *p* = 0.068, which met the pre-specified 1-sided α of 0.10) [13]. At the time of GRIFFIN final analysis, which occurred after all patients completed ≥1 year of long-term follow-up after the end of study treatment, death, or withdrawal (median follow-up, 49.6 months), responses continued to deepen over time for D-RVd versus RVd, and there was an improvement in progression-free survival (PFS) for the D-RVd group versus the RVd group (hazard ratio, 0.45; 95% confidence interval, 0.21–0.95; *p* = 0.032) [14]. The safety profiles of D-KRd and D-RVd were previously reported [11, 13]. No unexpected safety concerns occurred for these daratumumab-based quadruplet therapies, and adverse events in each regimen were consistent with previous reports of the individual regimen components.

Patients with MM may have high-risk disease characteristics, such as the presence of extramedullary disease, International Staging System stage III disease, advanced age, and/or the presence of high-risk cytogenetic abnormalities (HRCAs) [15–17]. These high-risk disease characteristics are associated with a poor overall prognosis and shorter survival, and patients with high-risk features constitute a population with high unmet medical need [15, 18, 19]. The consensus from the International Myeloma Working Group (IMWG) advises that cytogenetic risk should be evaluated using bone marrow aspirate–based fluorescence in situ hybridization panels for t(4;14), del(17p), and t(14;16), with an extended panel for clinical trials that includes t(11;14), t(14;20), gain(1q), del(1p), del(13q), and ploidy status [18]. While risk stratification is important for understanding overall prognosis, much remains to be learned about how HRCAs impact clinical outcomes and influence optimal therapy selection and treatment sequencing. The objective of this study is to better understand clinical outcomes for daratumumab-based treatment among patients with NDMM by HRCA risk stratifications, according to a revised definition inclusive of the cytogenetic abnormalities del(17p), t(4;14), t(14;16), t(14;20), and/or gain/amp(1q21) (≥3 copies of chromosome 1q21). Here, we present a post hoc analysis of side-by-side results including patients from MASTER (D-KRd) and GRIFFIN (D-RVd) with 0, 1, or ≥2 HRCAs, noting the goal is not to compare D-KRd and D-RVd but rather to evaluate the overall value of frontline daratumumab-based therapy.

## Methods

### Patients and study design

The full details of the MASTER (ClinicalTrials.gov Identifier: NCT03224507) [11] and GRIFFIN (ClinicalTrials.gov Identifier: NCT02874742) [13] studies have been previously reported. Briefly, in the multicenter, single-arm, phase 2 MASTER study, D-KRd was evaluated in transplant-eligible patients with NDMM. Patients had no upper age limit and an Eastern Cooperative Oncology Group performance status score of ≤2. Patients received up to 4 D-KRd induction cycles; high-dose therapy and ASCT; and up to 2 phases of D-KRd consolidation therapy (Cycles 5–8 and 9–12). MRD assessments in MASTER occurred post-induction, post-ASCT, and after each consolidation phase, and patients achieving 2 consecutive MRD negative (10^–5^) assessments transitioned to treatment-free observation. Patients who completed consolidation without 2 consecutive MRD-negative assessments transitioned to lenalidomide maintenance. The study design included enrichment for patients with MM harboring HRCAs to meet the criteria that ≥35% of participants would have t(4;14), t(14;16), and/or del(17p). In both studies, cytogenetic risk was assessed at baseline by fluorescence in situ hybridization via local testing.

In the multicenter, randomized, open-label, phase 2 GRIFFIN study, D-RVd was evaluated versus RVd alone in transplant-eligible patients with NDMM. Patients were 18–70 years of age and had an Eastern Cooperative Oncology Group performance status score of ≤2. Prior to the randomized phase of GRIFFIN, a safety run-in was conducted in 16 patients to assess D-RVd dose-limiting toxicities [20]. Following completion of the safety run-in, the study proceeded to the randomization phase in which patients were randomized 1:1 to the D-RVd or the RVd group. In this phase, patients received 4 D-RVd or RVd induction cycles, followed by high-dose therapy and ASCT, then 2 D-RVd or RVd consolidation cycles, followed by up to 2 years of maintenance therapy consisting of daratumumab plus lenalidomide or lenalidomide alone. Patients in the safety run-in phase of GRIFFIN received the same treatment as patients in the randomized phase in the D-RVd group. MRD negativity was measured at baseline, at first evidence of suspected CR or stringent CR (including patients with very good partial response or better and suspected daratumumab interference), after induction therapy, at the post-transplant consolidation disease evaluation, and after 1 and 2 years of maintenance therapy.

For both studies, the protocols and appropriate related documents were approved by the institutional review board or independent ethics committee at each participating site, and all patients gave written informed consent. The studies were conducted in accordance with the International Conference on Harmonisation Good Clinical Practice guidelines, the principles originating from the Declaration of Helsinki, as well as study site–specific regulations. The MASTER study followed the University of Alabama at Birmingham O’Neal Comprehensive Cancer Center Data and Safety Monitoring Plan. Each study established an independent Data Monitoring Committee for oversight for study conduct.

### Endpoints, objectives, and analyses

The primary endpoint of the MASTER study was the achievement of MRD negativity at any time during therapy and was previously reported [11]. The primary endpoint of GRIFFIN was the stringent CR rate by the end of post-ASCT consolidation treatment and was also previously published [13]. Both studies also assessed additional endpoints, including response rates, MRD-negativity rates (minimum sensitivity threshold of 1 in 100,000 cells [10^−5^]), and PFS. In GRIFFIN, response to study treatment and PFS were evaluated using a validated computer algorithm in alignment with IMWG criteria [21, 22]. Patients were considered MRD positive if the MRD assessment was positive, indeterminate, or unavailable. Best response on study for MASTER was also evaluated using IMWG criteria.

The objective of this post hoc analysis was to evaluate the clinical efficacy of the daratumumab-based quadruplet therapies D-KRd (from MASTER) and D-RVd (from GRIFFIN) in patients with NDMM with HRCAs, defined as having ≥1 of the following genetic abnormalities: del(17p), t(4;14), t(14;16), t(14;20), and/or gain/amp(1q21) (≥3 copies of chromosome 1q21). Cytogenetic abnormalities (fluorescence in situ hybridization [FISH]) were assessed by the local labs, normally accessed at the study sites, on bone marrow aspirates in both MASTER and GRIFFIN. Patients with evaluable data were grouped into standard risk, high risk, or ultra-high risk based on the presence of 0, 1, or ≥2 HRCAs, respectively. In addition, MRD-negativity rates were also presented for patients achieving ≥CR in each cytogenetic subgroup. This study descriptively presents the results for the D-KRd and D-RVd groups side by side, and thus no statistical or treatment comparisons between the 2 groups were performed. Kaplan–Meier plots and estimates of PFS were provided for each HRCA group in each study.

## Results

### Patient characteristics

A total of 123 patients with NDMM were enrolled in the MASTER study; most patients had ≥1 HRCA (1 HRCA, 37.4%, *n* = 46; ≥2 HRCAs, 19.5%, *n* = 24) and the remainder had 0 HRCA (43.1%, *n* = 53). There was no difference in the median duration of study treatment for D-KRd induction, ASCT, and consolidation among patients with NDMM with 0, 1, or ≥2 HRCAs, which was 11.5, 11.5, and 11.7 months, respectively (Table 1). Among 120 patients with NDMM in GRIFFIN who received D-RVd therapy (*n* = 104 randomized phase and *n* = 16 safety run-in), most had 0 HRCA (55.8%, *n* = 67) or 1 HRCA (28.3%, *n* = 34), and a smaller number of patients had ≥2 HRCAs (10.8%, *n* = 13). Six (5.0%) GRIFFIN patients were not evaluable for cytogenetic abnormalities because cytogenetic testing was not done or data were not captured; therefore, these patients were not included in this analysis. The median duration of study treatment for D-RVd induction, ASCT, and consolidation was 8.1, 8.1, and 7.4 months among patients with NDMM with 0, 1, or ≥2 HRCAs, respectively, and the median duration of study maintenance therapy was 24.4, 24.2, and 23.9 months for patients with 0, 1, or ≥2 HRCAs, respectively (Table 2). In both MASTER and GRIFFIN, the median age of patients was 60 years. Among patients with ≥2 HRCAs, a relatively high proportion had International Staging System stage III disease (45.8% [*n* = 11] of D-KRd patients and 30.8% [*n* = 4] of D-RVd patients) or extramedullary disease (7.7% [*n* = 1] of D-RVd; data on extramedullary disease were not available in MASTER).

**Table 1:** Baseline characteristics for patients who received D-KRd in MASTER.

Characteristic	D-KRd			
	0 HRCA *n* = 53	1 HRCA *n* = 46	≥2 HRCAs *n* = 24	Total *n* = 123
Median age (range), years	60 (36–79)	61 (35–77)	60 (41–72)	60 (35–79)
Sex, *n* (%)				
Male	33 (62.3)	24 (52.2)	13 (54.2)	70 (56.9)
Female	20 (37.7)	22 (47.8)	11 (45.8)	53 (43.1)
ISS disease stage, *n* (%)^a^				
I	28 (52.8)	15 (32.6)	5 (20.8)	48 (39.0)
II	20 (37.7)	19 (41.3)	8 (33.3)	46 (37.4)
III	5 (9.4)	12 (26.1)	11 (45.8)	29 (23.6)
Cytogenetic abnormality, *n* (%)^b^				
del(17p)	0	12 (26.1)	14 (58.3)	26 (21.1)
t(4;14)	0	8 (17.4)	13 (54.2)	21 (17.1)
t(14;16)	0	2 (4.3)	4 (16.7)	6 (4.9)
Gain/amp(1q21)	0	24 (52.2)	20 (83.3)	44 (35.8)
t(14;20)	0	0	0	0
Median duration of study treatment, months				
Induction/consolidation^c^	11.5	11.5	11.7	11.5

*ASCT* autologous stem cell transplant, *D-KRd* daratumumab plus carfilzomib/lenalidomide/dexamethasone, *HRCA* high-risk cytogenetic abnormality, *ISS* International Staging System.

^a^ISS disease stage is based on the combination of serum β_2_-microglobulin and albumin levels. Higher stages indicate more advanced disease.

^b^Cytogenetic risk was assessed by fluorescence in situ hybridization (local testing).

^c^Duration of study treatment is from onset of therapy to completion of consolidation therapy, including ASCT.

**Table 2:** Baseline characteristics for patients who received D-RVd in GRIFFIN.

Characteristic	D-RVd^a^			
	0 HRCA *n* = 67	1 HRCA *n* = 34	≥2 HRCAs *n* = 13	Total *n* = 114
Median age (range), years	59.0 (34–70)	59.5 (29–70)	62.0 (49–70)	60.0 (29–70)
Sex, *n* (%)				
Male	37 (55.2)	18 (52.9)	9 (69.2)	64 (56.1)
Female	30 (44.8)	16 (47.1)	4 (30.8)	50 (43.9)
ISS disease stage, *n* (%)^b^				
I	42 (62.7)	13 (38.2)	5 (38.5)	60 (52.6)
II	20 (29.9)	17 (50.0)	4 (30.8)	41 (36.0)
III	5 (7.5)	4 (11.8)	4 (30.8)	13 (11.4)
Cytogenetic abnormality, *n* (%)^c^				
del(17p)	0	4 (11.8)	8 (61.5)	12 (10.5)
t(4;14)	0	3 (8.8)	5 (38.5)	8 (7.0)
t(14;16)	0	0	1 (7.7)	1 (0.9)
Gain/amp(1q21)	0	26 (76.5)	12 (92.3)	38 (33.3)
t(14;20)	0	1 (2.9)	0	1 (0.9)
Median duration of study treatment, months^d^				
Induction/consolidation^e^	8.1	8.1	7.4	8.1
Maintenance	24.4	24.2	23.9	24.2

*ASCT* autologous stem cell transplant, *D-RVd* daratumumab plus lenalidomide/bortezomib/dexamethasone, *HRCA* high-risk cytogenetic abnormality, *ISS* International Staging System.

^a^For GRIFFIN, the D-RVd group included patients from the randomized phase (*n* = 104) and the safety run-in phase (*n* = 16). Patients were grouped by HRCA: 0 HRCA (*n* = 67), 1 HRCA (*n* = 34), or ≥2 HRCAs (*n* = 13). 6 patients were not evaluable for cytogenetic abnormalities.

^b^ISS disease stage is based on the combination of serum β_2_-microglobulin and albumin levels. Higher stages indicate more advanced disease.

^c^Cytogenetic risk was assessed by fluorescence in situ hybridization (local testing).

^d^Study duration is reported for treated patients for induction/consolidation (0 HRCA, *n* = 66; 1 HRCA, *n* = 32; ≥2 HRCAs, *n* = 13; total, *n* = 111) and maintenance (0 HRCA, *n* = 62; 1 HRCA, *n* = 29; ≥2 HRCAs, *n* = 10; total, *n* = 101).

^e^Duration of study treatment is from initiation of therapy to completion of consolidation therapy, including ASCT.

### Efficacy

**Fig. 1:**
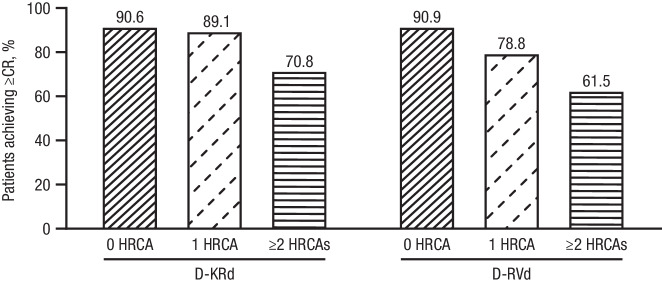
Rates of ≥CR (best response on study) by cytogenetic risk status* among patients who received D-KRd in MASTER^†^ and D-RVd in GRIFFIN^‡^. Rates of ≥CR were assessed based on International Uniform Response Criteria Consensus Recommendations, and percentages were calculated with the number of patients in the treatment group as the denominator. ≥CR complete response or better, D-KRd daratumumab plus carfilzomib/lenalidomide/dexamethasone, D-RVd daratumumab plus lenalidomide/bortezomib/dexamethasone, HRCA high-risk cytogenetic abnormality. *HRCAs include any of the following genetic abnormalities: del(17p), t(4;14), t(14;16), t(14;20), and gain/amp(1q21) (≥3 copies of chromosome 1q21). Patients were grouped into categories: standard risk (0 HRCA), high risk (1 HRCA), or ultra-high risk (≥2 HRCAs). ^†^Evaluable patients in MASTER included all enrolled patients (0 HRCA, *n* = 53; 1 HRCA, *n* = 46; ≥2 HRCAs, *n* = 24). ^‡^Evaluable patients in GRIFFIN were the response-evaluable population (0 HRCA, *n* = 66; 1 HRCA, *n* = 33; ≥2 HRCAs, *n* = 13).

In an analysis of best response at any time point during the study, rates of ≥CR were highest for patients with 0 or 1 HRCA in both GRIFFIN and MASTER (Fig. 1). In MASTER, rates of ≥CR among D-KRd patients were 90.6%, 89.1%, and 70.8% for 0, 1, or ≥2 HRCAs, respectively. In GRIFFIN, rates of ≥CR among D-RVd patients were 90.9%, 78.8%, and 61.5% for 0, 1, or ≥2 HRCAs.

MRD-negativity (both 10^–5^ and 10^–6^) rates were generally similar for D-KRd across patients with 0, 1, or ≥2 HRCAs, but were highest for D-KRd in patients with 1 HRCA (Table 3). In MASTER, MRD-negativity rates following D-KRd were 80.0%, 86.4%, and 83.3% at the 10^–5^ threshold for 0, 1, or ≥2 HRCAs, respectively, and 68.0%, 79.5%, and 66.7% at the 10^–6^ threshold. In D-KRd patients who achieved ≥CR, MRD-negativity (10^–5^) rates were 84.4%, 89.7%, and 94.1% for 0, 1, or ≥2 HRCAs, respectively. Rates of sustained MRD negativity (10^–5^) lasting ≥12 months were 64.0%, 72.7%, and 50.0% among D-KRd patients with 0, 1, or ≥2 HRCAs. Median time to MRD negativity (10^–5^) was similar across HRCA groups (0 HRCA, 7.5 months; 1 HRCA, 7.1 months; ≥2 HRCAs, 7.6 months). In GRIFFIN, MRD-negativity (10^–5^) rates in D-RVd patients were 76.1%, 55.9%, and 61.5% for 0, 1, or ≥2 HRCAs, respectively. MRD-negativity (10^–6^) rates were higher for D-RVd patients with 0 HRCA (44.8%) and 1 HRCA (26.5%) compared with ≥2 HRCAs (15.4%). In patients who achieved ≥CR, MRD-negativity (10^–5^) rates in D-RVd patients were 83.3%, 69.2%, and 87.5% among patients with 0, 1, or ≥2 HRCAs, respectively. Rates of sustained MRD negativity (10^–5^) lasting ≥12 months were 53.7%, 38.2%, and 30.8% among D-RVd patients with 0, 1, or ≥2 HRCAs. Median time to MRD negativity (10^–5^) was 8.5, 8.6, and 19.6 months among D-RVd patients with 0, 1, or ≥2 HRCAs.

**Table 3:** MRD negativity by cytogenetic risk status^a^ among patients who received D-KRd in MASTER and D-RVd in GRIFFIN.

	D-KRd			D-RVd		
	0 HRCA	1 HRCA	≥2 HRCAs	0 HRCA	1 HRCA	≥2 HRCAs
MRD negative						
Evaluable population	*n* = 50^b^	*n* = 44^b^	*n* = 24^b^	*n* = 67^c^	*n* = 34^c^	*n* = 13^c^
10^–5^ sensitivity, %	80.0	86.4	83.3	76.1	55.9	61.5
10^–6^ sensitivity, %	68.0	79.5	66.7	44.8	26.5	15.4
In patients achieving ≥CR	*n* = 45	*n* = 39	*n* = 17	*n* = 60	*n* = 26	*n* = 8
10^–5^ sensitivity, %	84.4	89.7	94.1	83.3	69.2	87.5
Durable MRD negativity lasting ≥12 months						
Evaluable population	*n* = 50^b^	*n* = 44^b^	*n* = 24^b^	*n* = 67^c^	*n* = 34^c^	*n* = 13^c^
10^–5^ sensitivity, %	64.0	72.7	50.0	53.7	38.2	30.8
MRD (10^–5^) conversion rate						
Evaluable population				*n* = 67^c^	*n* = 34^c^	*n* = 13^c^
MRD positive by the end of induction and then became MRD negative, %	NA	NA	NA	49.3	41.2	38.5
MRD positive by the end of consolidation and then became MRD negative, %	NA	NA	NA	19.4	11.8	23.1
Median time to MRD (10^–5^) negativity,^b,c^ months	7.5	7.1	7.6	8.5	8.6	19.6

*MRD* minimal residual disease, *D-KRd* daratumumab plus carfilzomib/lenalidomide/dexamethasone, *D-RVd* daratumumab plus lenalidomide/bortezomib/dexamethasone, *HRCA* high-risk cytogenetic abnormality, *CR* complete response, *NA* not available.

^a^HRCAs include any of the following genetic abnormalities: del(17p), t(4;14), t(14;16), t(14;20), and gain/amp(1q21) (≥3 copies of chromosome 1q21). Patients were grouped into categories: standard risk (0 HRCA), high risk (1 HRCA), or ultra-high risk (≥2 HRCAs).

^b^For MASTER, data are for all enrolled patients with available MRD data.

^c^For GRIFFIN, the D-RVd group included patients from the randomized phase (*n* = 104) and the safety run-in phase (*n* = 16). Patients were grouped by HRCA: 0 HRCA (*n* = 67), 1 HRCA (*n* = 34), or ≥2 HRCAs (*n* = 13). 6 patients were not evaluable for cytogenetic abnormalities.

**Fig. 2:**
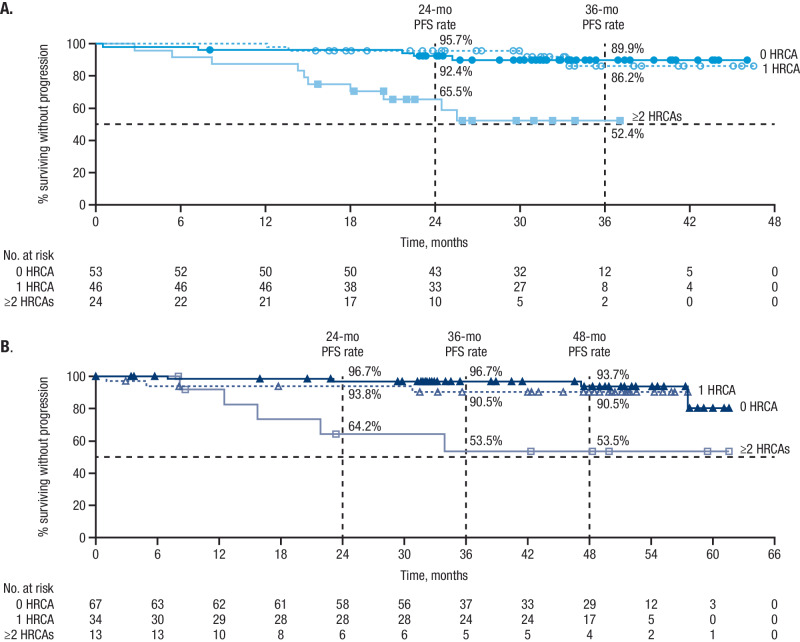
PFS by cytogenetic risk status* among patients who received D-KRd in MASTER and D-RVd in GRIFFIN. Kaplan–Meier estimates of PFS among patients in the ITT population by cytogenetic risk status (0 HRCA, 1 HRCA, or ≥2 HRCAs) are shown for (**A**) MASTER and (**B**) GRIFFIN. Median PFS was not reached for any group. PFS progression-free survival, D-KRd daratumumab plus carfilzomib/lenalidomide/dexamethasone, D-RVd daratumumab plus lenalidomide/bortezomib/dexamethasone, HRCA high-risk cytogenetic abnormality. *HRCAs include any of the following genetic abnormalities: del(17p), t(4;14), t(14;16), t(14;20), and gain/amp(1q21) (≥3 copies of chromosome 1q21). Patients were grouped into categories: standard risk (0 HRCA), high risk (1 HRCA), or ultra-high risk (≥2 HRCAs).

PFS rates in MASTER and GRIFFIN were superior for patients with 0 or 1 HRCA compared with ≥2 HRCAs (Fig. 2). In MASTER, at a median follow-up of 31.1 months, estimated 24-month PFS rates for D-KRd patients were 92.4%, 95.7%, and 65.5% for 0, 1, or ≥2 HRCAs, respectively, and estimated 36-month rates were 89.9%, 86.2%, and 52.4%. In GRIFFIN, PFS analyses were conducted for D-RVd patients in a combined analysis of randomized patients (median follow-up at final analysis among all randomized patients, 49.6 months) and among patients from the safety run-in phase (median follow-up, 59.5 months). Estimated 36-month rates for all D-RVd patients were 96.7%, 90.5%, and 53.5% for 0, 1, or ≥2 HRCAs, respectively, and estimated 48-month PFS rates were 93.7%, 90.5%, and 53.5%.

In MASTER, 3 patients died while on D-KRd study therapy (0 HRCA, *n* = 2 [both from sudden death]; ≥2 HRCAs, *n* = 1 [metapneumovirus during transplant]). After discontinuation of D-KRd therapy and during follow-up, 2 patients died, but neither death was preceded by progressive disease (PD; 1 HRCA, *n* = 2 [fall and COVID-19 pneumonia]). In GRIFFIN, 8 D-RVd patients died (0 HRCA, *n* = 2 [due to an adverse event (bronchopneumonia) and PD]; 1 HRCA, *n* = 1 [due to PD]; ≥2 HRCAs, *n* = 4 [all due to PD]; not evaluable for cytogenetics, *n* = 1 [due to respiratory failure]).

## Discussion

This analysis of transplant-eligible patients with NDMM by cytogenetic risk status from the MASTER and GRIFFIN studies showed that patients with high cytogenetic risk derive clinical benefit from frontline daratumumab-based quadruplet therapy. In both MASTER and GRIFFIN, patients with 0 or 1 HRCA achieved higher rates of ≥CR than patients with ≥2 HRCAs. PFS results by cytogenetic risk were similar between the MASTER and GRIFFIN studies and showed high estimated PFS rates for patients with 0 or 1 HRCA for both D-KRd and D-RVd therapy; these were superior to PFS rates for patients with ≥2 HRCAs. In general, rates of MRD negativity and durable MRD negativity were highest among patients with 0 or 1 HRCA, similar to the results for ≥CR and PFS. Together, these observations indicate that patients with standard-risk and high-risk disease (≤1 HRCA) respond well to frontline daratumumab-based quadruplet therapy, specifically to MRD-directed therapy in MASTER, and have similar outcomes. In fact, it is remarkable that the outcomes of patients with 1 HRCA in the setting of quadruplet induction/consolidation and ASCT greatly resemble the outcomes of patients with standard-risk disease. It should also be noted that, in both studies, all patients had improvement in clinical outcomes by MRD assessment following ASCT, sustaining the benefit of this portion of the treatment regimens. However, the relatively rapid progression seen among patients with >2 HRCAs in both studies indicates that innovations beyond the addition of CD38 antibody therapy are needed to improve outcomes among ultra-high−risk patients (≥2 HRCAs). In aggregate, this observation is a testament to the notion that risk is context dependent and potentially modifiable by therapy.

It has been established that MM patients with HRCAs generally have a worse prognosis, and that the prognosis of these patients varies depending on a number of factors, including the presence and the amount of specific genetic abnormalities as well as choice of therapy [18]. To this end, there is considerable interest in understanding whether emerging MM therapies provide clinical benefit among patients with HRCAs and how upfront therapy can be optimally used to target high-risk groups. Several phase 3 clinical trials of daratumumab-based regimens among patients with NDMM have shown that the addition of daratumumab improves outcomes for patients with high-risk cytogenetics compared with non–daratumumab-based standard-of-care regimens [23–25]. A meta-analysis including these randomized studies of daratumumab (MAIA [daratumumab plus lenalidomide and dexamethasone], ALCYONE [daratumumab plus bortezomib, melphalan, and prednisone], and CASSIOPEIA [daratumumab plus bortezomib, thalidomide, and dexamethasone]) in patients with NDMM with high cytogenetic risk further validated the outcomes reported in the individual studies. Specifically, Giri et al. demonstrated that the addition of daratumumab to standard-of-care regimens reduced the risk of disease progression or death by 33% for patients with high-risk disease versus standard of care alone (hazard ratio, 0.67; 95% confidence interval, 0.47–0.95) [26]. To be noted, the methods for the assessment of high-risk MM varied across the included studies. For ALCYONE and MAIA, high-risk patients were defined as having ≥1 of the following HRCAs at baseline: del(17p), t(4;14), and t(14;16) [23, 24]. For CASSIOPEIA, high-risk MM was defined as having del(17p) and/or t(4;14), without the inclusion of t(14;16) [25]. A pooled analysis of patient-level data from MAIA and ALCYONE, which included transplant-ineligible patients with high-risk NDMM defined as having del(17p), t(4;14), and/or t(14;16), demonstrated that, with a median follow-up of 43.7 months, daratumumab-based therapy reduced the risk of disease progression or death by 41% (adjusted hazard ratio, 0.59; 95% confidence interval, 0.41–0.85) versus the standard-of-care regimen without daratumumab (bortezomib, melphalan, and prednisone or lenalidomide and dexamethasone) [27]. In this analysis, the estimated proportion of patients who did not progress and were still alive at 36 months was 41.3% among those who received daratumumab-based therapy versus 19.9% among those who received standard of care [27]. Our present report of HRCAs from the MASTER and GRIFFIN studies includes transplant-eligible patients with NDMM who received daratumumab-based quadruplet therapy plus ASCT, and HRCAs were defined according to a newer, revised definition (≥1 of the following: del[17p], t[4;14], t[14;16], t[14;20], and/or gain/amp[1q21]).

In this analysis, we performed post hoc analyses by the number of HRCAs (0, 1, or ≥2). As noted, ≥CR rates and PFS were similar for patients with 0 or 1 HRCA and worse for patients with ≥2 HRCAs; however, this should be interpreted in the context that the groups were not stratified (eg, by revised International Staging System). Additionally, in this study, gain/amp(1q21) accounted for a relatively high proportion of patients with only 1 HRCA in both the D-KRd (52.2%) and D-RVd (76.5%) groups. This analysis does not evaluate individual HRCAs as the number of patients with any specific HRCA is very limited. Other studies have demonstrated that the number and type of HRCA can be an indicator of prognosis. An analysis of patients with NDMM in the Medical Research Council (MRC) Myeloma IX trial [28] who received cyclophosphamide, vincristine, doxorubicin, and dexamethasone or cyclophosphamide, thalidomide, and dexamethasone followed by transplant showed that the presence of ≥2 chromosomal abnormalities (defined as t[4;14], t[14;16], t[14;20], gain[1q], and/or del[17p]) was associated with more aggressive disease [29]. In this analysis, median PFS was 30.8, 21.9, and 14.4 months for patients with 0, 1, and ≥2 HRCAs, respectively [29]. Acknowledging the limitations of cross-study comparisons (eg, differences in study designs and treatment backbones), it is notable that the poor prognosis of patients with ≥2 HRCAs seen in GRIFFIN and MASTER is consistent with the findings from the MRC Myeloma IX study. Interestingly, patients with 0 and 1 HRCA who received daratumumab-based quadruplet therapy in GRIFFIN or MASTER had similar PFS outcomes, which contrasts with the MRC Myeloma IX study, where patients with 1 HRCA had better intermediate PFS outcomes than those with ≥2 HRCAs, but not as high as those with 0 HRCA. It should also be considered that PFS outcomes overall were lower in the MRC Myeloma IX study versus PFS outcomes in MASTER and GRIFFIN. Our observations from this combined MASTER and GRIFFIN analysis showed that daratumumab-based quadruplet therapy had similar efficacy among patients with 0 and 1 HRCA. Further and more in-depth investigations of the optimal use of daratumumab-based therapy in high-risk transplant-eligible patients with NDMM, based on the number and identity of specific HRCAs, are warranted. For patients with ultra-high−risk disease, promising outcomes were observed in the OPTIMUM/MUKnine study, which evaluated frontline daratumumab-containing induction and extended/intensified consolidation with transplant for patients with ≥2 of the following: t(4;14), t(14;16), t(14;20), gain(1q), del(1p), del(17p); or gene expression SKY92 (SkylineDx, Rotterdam, The Netherlands) profiling; or with primary plasma cell leukemia (circulating plasma cells >20%) [30].

It is important to note that the study designs of MASTER and GRIFFIN are different, so direct comparisons should be avoided. Neither MASTER nor GRIFFIN collected information on clone size for individual HRCAs or copy number for 1q21 abnormalities. Given the potential heterogeneity in cut-offs used to determine the presence of cytogenetic abnormalities across local laboratories, this lack of information on clone size may be a notable limitation. Per the recommendations of the European Myeloma Network [31], the appropriate cut-off level is 10% for fusion abnormalities and 20% for numerical abnormalities. Additionally, 1q21 abnormalities were assessed as a single "gain/amp(1q21)" group, without further differentiation, making it difficult to assess the prognostic value of gain 1q21 versus the more prognostic adverse amplification of 1q21. Additionally, MASTER provided enrichment for patients with HRCAs, while GRIFFIN did not. The goal of this analysis was not to compare D-KRd and D-RVd treatments, but rather to evaluate the overall value of frontline daratumumab-based treatment. The results reported here demonstrate that daratumumab-based quadruplet frontline therapy with ASCT provides clinical benefit to patients with ≤1 HRCA, but new approaches are needed for patients with ≥2 HRCAs, which is an area of significant unmet need. It will be necessary to explore the use of novel agents and treatment combinations for this group, including bispecific T-cell engagers and chimeric antigen receptor T cells. In addition, prolonged or indefinite multiagent maintenance strategies may be required to extend PFS in such patients. In summary, continued research, longer follow-up, and larger studies are needed to fully understand and identify the optimal therapies for patients with HRCAs, especially for patients with ultra-high−risk disease (≥2 HRCAs).

## Data Availability

For the GRIFFIN study, the data sharing policy of Janssen Pharmaceutical Companies of Johnson & Johnson is available at https://www.janssen.com/clinical-trials/transparency. As noted on this site, requests for access to the study data can be submitted through Yale Open Data Access (YODA) Project site at http://yoda.yale.edu. For the investigator-initiated MASTER study, although these data are not currently publicly available, requests for sharing can be sent to the corresponding author of this manuscript and will be evaluated on an individual basis.
